# Achieving λ/10 Resolution CW STED Nanoscopy with a Ti:Sapphire Oscillator

**DOI:** 10.1371/journal.pone.0040003

**Published:** 2012-06-27

**Authors:** Yujia Liu, Yichen Ding, Eric Alonas, Wenli Zhao, Philip J. Santangelo, Dayong Jin, James A. Piper, Junlin Teng, Qiushi Ren, Peng Xi

**Affiliations:** 1 School of Biomedical Engineering, Shanghai Jiao Tong University, Shanghai, China; 2 Department of Biomedical Engineering, College of Engineering, Peking University, Beijing, China; 3 Wallace H Coulter Department of Biomedical Engineering, Georgia Institute of Technology and Emory University, Atlanta, Georgia, United States of America; 4 Advanced Cytometry Labs, MQphotonics Research Centre, Macquarie University, Sydney, New South Wales, Australia; 5 College of Life Sciences, Peking University, Beijing, China; German Cancer Research Center, Germany

## Abstract

In this report, a Ti:Sapphire oscillator was utilized to realize synchronization-free stimulated emission depletion (STED) microscopy. With pump power of 4.6 W and sample irradiance of 310 mW, we achieved super-resolution as high as 71 nm. With synchronization-free STED, we imaged 200 nm nanospheres as well as all three cytoskeletal elements (microtubules, intermediate filaments, and actin filaments), clearly demonstrating the resolving power of synchronization-free STED over conventional diffraction limited imaging. It also allowed us to discover that, Dylight 650, exhibits improved performance over ATTO647N, a fluorophore frequently used in STED. Furthermore, we applied synchronization-free STED to image fluorescently-labeled intracellular viral RNA granules, which otherwise cannot be differentiated by confocal microscopy. Thanks to the widely available Ti:Sapphire oscillators in multiphoton imaging system, this work suggests easier access to setup super-resolution microscope via the synchronization-free STED.

## Introduction

Fluorescence microscopy has become an essential tool to study biological molecules, pathways and events in living cells, tissues and animals. Its key advantages over other forms of microscopy arise from the use of specific, minimally invasive molecular staining and compatibility with living cells. Fluorescence microscopes with the most advanced confocal geometries available yield optical resolution approaching the theoretical Abbe diffraction limit of ∼ 200 nm, but this is still larger than many subcellular structures, which are too small to be observed in detail. These limitations have driven the development of super-resolution optical imaging methodologies over the past decade, and have significantly impacted many fields of science [1], [2], [3], [4], [5].

Bypassing the optical diffraction limit in far-field optical microscopy has been realized by two key pioneering concepts: spatially patterned excitation with emission inhibition, such as Stimulated Emission Depletion (STED) microscopy [6], [7], [8] and Saturated Structured Illumination Microscopy (SSIM) [9], [10]; and high-precision localization of single fluorophores through individual activation, such as STochastic Optical Reconstruction Microscopy (STORM) [11], [12] and Photo-Activated Localization Microscopy (PALM) [13]. Stimulated Emission Depletion (STED) was the first and most direct approach to overcoming the diffraction limit for far-field nanoscopy. STED microscopy uses an intense doughnut-shaped beam surrounding the excitation focus to switch the fluorophore(s) in the sample to a “dark” state through stimulated emission. This effectively eliminates the periphery of the Point Spread Function (PSF), resulting in a narrower PSF, or super-resolution.

The depletion efficiency of STED can be optimized with the pulsed excitation and the temporal delayed pulsed STED beam [7]. This requires precise synchronization of two laser pulses and maintenance of the accurate time delay [14], [15], [16], [17], [18], [19], [20]. To meet these requirements, either a laser diode was specially designed with a rather sophisticated optical or electrical delay line [14], [15], [16], [17], [18], or a single laser was used to generate both excitation and STED pulses to minimize synchronization steps [19], [20]. Continuous Wave (CW) STED, with both excitation and STED beams in CW mode, can be engineered based on spectral discrimination of the spontaneous emission and stimulated emission of the fluorescent molecules, and has been successfully applied to image neurofilaments in neuroblastoma cells [21], syntaxin clusters in the cell membrane [21], the nuclear lamina of mammalian cells [21], and the endoplasmic reticulum of living cells [22]. CW STED is beneficial because it is synchronization-free, and requires no pulse length or timing optimization for the excitation and depletion beams. This characteristic makes it easier to adapt to a conventional confocal fluorescence configurations. Previous reports of CW STED utilized either high pump power laser systems [21], or the combination of multiple fiber lasers [22]. However, these lasers are not readily available in biomedical instrumentation labs, which might explain their limited use. The only other current option for CW STED are commercial microscopes, but these are prohibitively expensive for most users.

On the other hand, the Ti:Sapphire laser has recently been used as both an excitation and depletion source in a two-photon STED system for super-resolution neuron imaging [23]; as well as for single-laser, single-wavelength STED nanoscopy [24]. The work presented here further expands the application of Ti:Sapphire lasers to STED nanoscopy.

In this paper, we demonstrated a synchronization-free CW STED microscope using a 6 Watt Diode-Pumped Solid State (DPSS) 532 nm laser pumped Ti:Sapphire oscillator tuned to CW mode, which is a source broadly available in many two-photon microscope systems [25], [26], [27], [28], [29]. Taking advantage of the wavelength tunability of the Ti:Sapphire laser, the depletion wavelength was optimized for different dyes. Using this system we demonstrated a super-resolution of 71 nm, and resolved the fine structures of actin filaments, intermediate filaments, and microtubules. In situations where optical deconvolution fails to interpret the structure of aggregated nanospheres, CW STED can correctly discern the nanospheres with ∼200 nm distance. Interestingly, a pair of parallel actin filaments, 240 nm from each other, was clearly imaged with STED, which could be misinterpreted as with a double-helix structure in confocal microscopy due to the lack of sufficient resolution. Furthermore, we applied the CW STED for the first time to visualize the viral genomic RNA of human respiratory syncytial virus (hRSV) in HEp-2 cells resulting in a high-resolution image clearly showing individual RNA granules. Moreover, we demonstrated the utility of Dylight 650 as a far-red dye suitable for STED microscopy with improved depletion efficiency.

## Results

**Figure 1:**
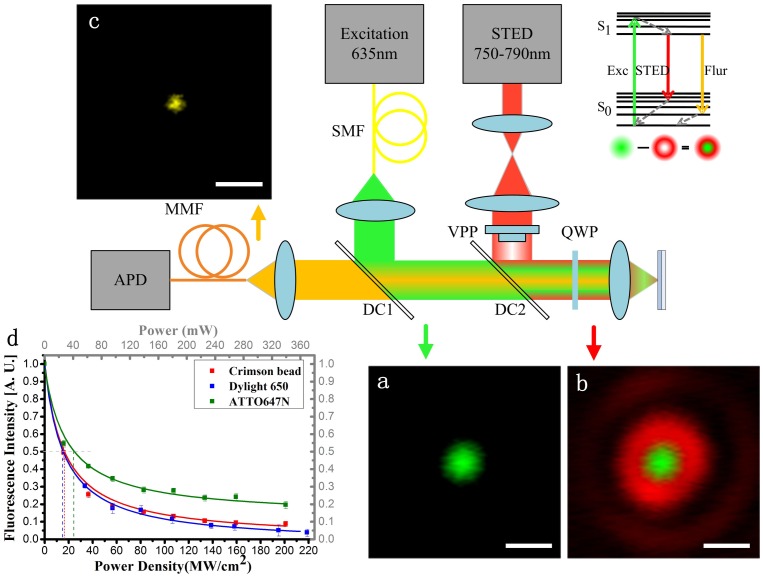
Schematic of the CW STED system for far-field super-resolution optical nanoscopy. The green line represents the excitation 635 nm laser, red line is the CW Ti: Sapphire STED beam, and the yellow line represents the fluorescence signal. SMF: single mode fiber; MMF: multi-mode fiber; DC1 and DC2 are dichotic filters. VPP: vortex 0–2π phase plate; QWP: quarter waveplate. The excitation PSF (a), doughnut depletion PSF overlapped with excitation PSF (b), and STED PSF (c) clearly shows the process of achieving super-resolution. Scale bar: 500 nm. The modulation efficiency versus the depletion intensity was measured with crimson beads, DyLight 650 and ATTO 647N solution (d). The STED wavelengths are 763 nm for crimson beads and ATTO 647N, and 783 nm for DyLight 650.

Figure 1 illustrates the schematic setup and preliminary evaluation of the stimulated emission depletion efficiencies on different fluorescent dyes (1d). We employed a vortex 0–2π phase plate to generate the doughnut shaped depletion beam for de-exciting the fluorescence by stimulated emission [30]. As the resolution is determined by the depletion efficiency, not the diffraction limit, we first studied the effect of the depletion ratio 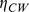 on the spontaneous fluorescence by overlapping the STED focus (without phase modulation) with the excitation focus [31]. As shown in Figure 1d, the saturation intensity 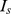 (in which the possibility of spontaneous emission and stimulated emission is equal) is measured to be ∼17 MW/cm^2^ for crimson beads at 763 nm, ∼14.4 MW/cm^2^ for DyLight 650 at 783 nm and ∼23.8 MW/cm^2^ for ATTO 647N at 763 nm (the intensities are measured before the 100x objective). The saturation intensity for ATTO647N is larger than previous literatures [21], [24], which may due to residual system optical aberration as well as the measurement condition, to which the extinction ratio of 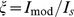 is insensitive. The reciprocal fitting of 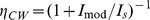 agrees well with the experimental data. When the STED beam was switched off, the system functioned as a normal confocal system, which was used to obtain data for comparison.

**Figure 2:**
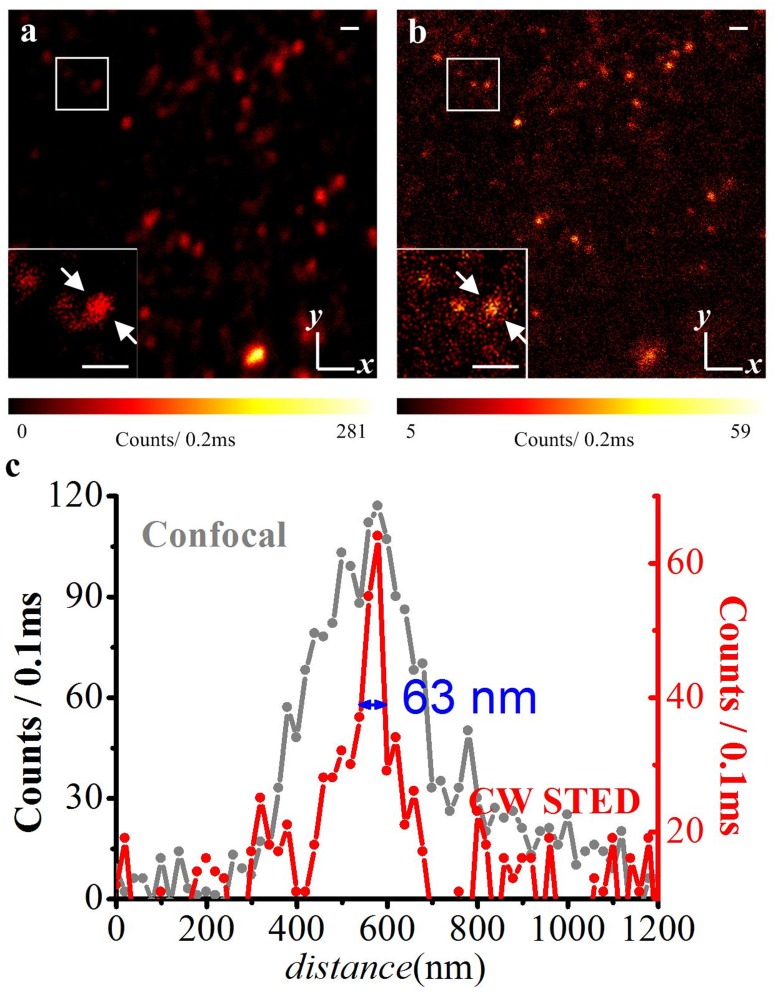
Confocal and corresponding CW-STED images of fluorescent 20-nm-diameter nanospheres. (a) Confocal; (b) CW-STED; (c) The profile along the dashed line in (a) and (b) exhibits a spot size of 63 nm, indicating an effective resolution of ∼59 nm after subtraction of the bead size. Scale bar: 500 nm.

The resolution of the prototype STED system was evaluated by imaging 20 nm crimson nanospheres. As shown Figure 2, at the maximum available STED power of 340 mW, the FWHM spot size was measured as small as 63 nm laterally, indicating an effective resolution of 59 nm after accounting for the size of the nanosphere [21]. With a total 20 nanospheres measured in the focal plane, the average resolution was calculated as 71 nm ±9 nm. This is in good agreement with the theoretical prediction of 
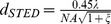
. The pixel dwell time is set to 0.2 ms in all the presented images.

**Figure 3:**
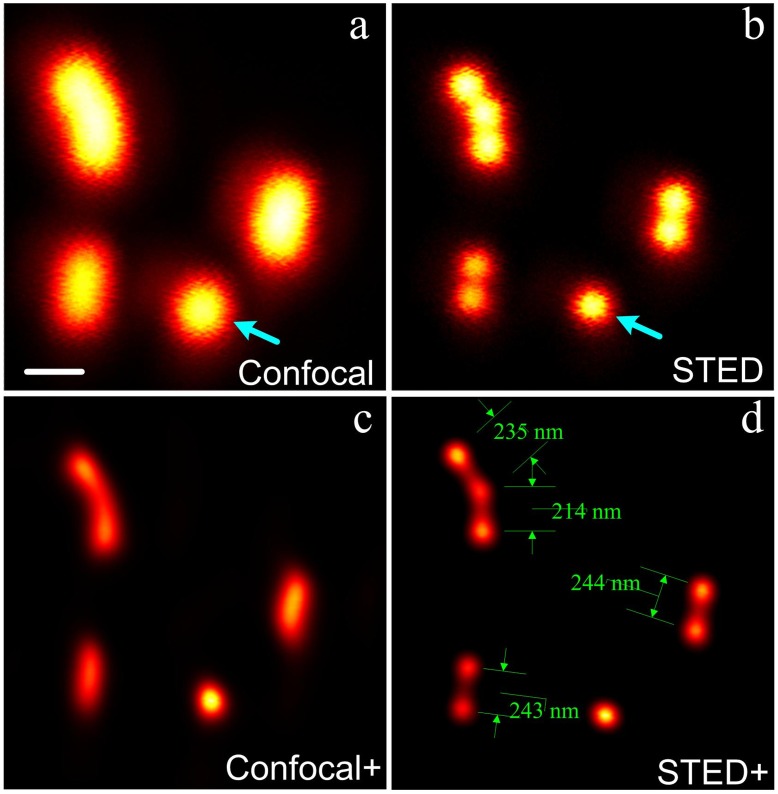
Comparison of diffraction-limited confocal microscopy and STED nanoscopy for fluorescent 200-nm-diameter nanospheres. (a) Confocal and (b) corresponding CW-STED images; (c) and (d) are the deconvolved results of (a) and (b). Scale bar: 500 nm.

Figure 3 shows images of the 200 nm nanospheres on glass. Adjacent nanospheres under the confocal system (a and c) can be clearly discriminated by the super-resolution CW STED system (b and d). Though the Richardson-Lucy (R-L) deconvolution algorithm can improve the resolution of both confocal and STED images (denoted confocal+ in a versus b, and STED+ in c versus d), once the adjacent nanospheres are within the optical diffraction limit, the STED+ (Fig 3d) can discern all the spheres clearly.

**Figure 4:**
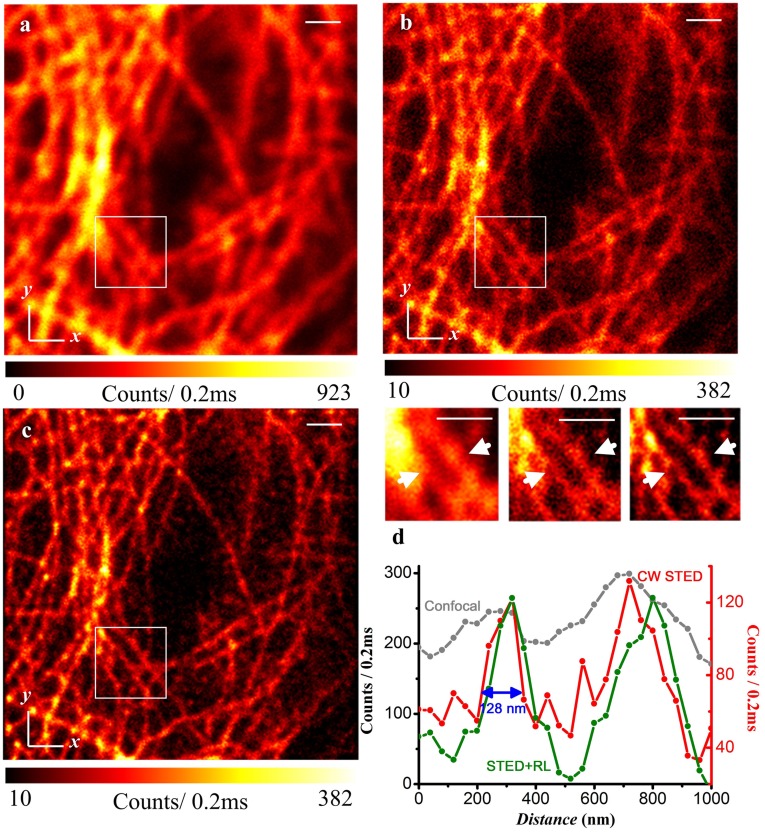
Confocal, corresponding CW-STED, and deconvolved STED images of microtubules in a Vero cell stained with Dylight 650. (a) Confocal; (b) CW-STED; (c) R-L deconvolution of (b). The intensity at the cross-section marked in (a), (b), and (c) inlets are plotted in (d). The wavelength and power density for the STED beam is 783 nm and ∼200 MW/cm^2^. Scale bar: 1 µm.

**Figure 5:**
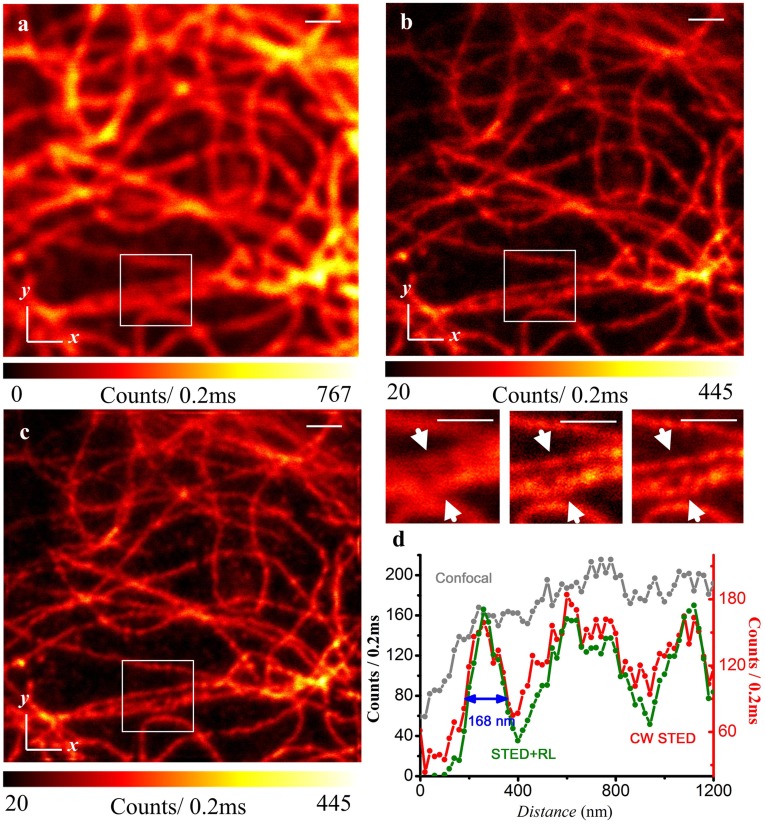
Confocal, corresponding CW-STED, and deconvolved STED images of keratin intermediate filaments in a PtK2 cell stained with ATTO 647N. (a) Confocal; (b) CW-STED; (c) R-L deconvolution of (b). The intensity at the cross-section marked in (a), (b), and (c) boxed regions are plotted in (d). The wavelength and power density for STED beam is 763 nm and ∼200 MW/cm^2^. Scale bar: 1 µm.

To demonstrate the resolving power of the CW STED nanoscope, all three cytoskeleton filaments were imaged: microtubules, intermediate filaments (keratin), and actin filaments. From the microtubule network of Vero cells, we can see that STED nanoscopy provides ∼128 nm FWHM with Dylight 650 staining (Figure 4). Because of the size of the microtubules is generally ∼25 nm, its junctions are hardly resolved with conventional microscope. However, with the STED, the fine microstructure can be visualized in Figure 4b, which result can be further improved by the R-L deconvolution in Figure 4c. In comparison, Figure 5 showed the images of the intermediate filament network in PtK2 cells, a male rat kangaroo kidney epithelial cell line stained with ATTO647N. The cross-sectional diameter is measured to be 168 nm on STED. The typical diameter of intermediate filament is ∼10 nm.

**Figure 6:**
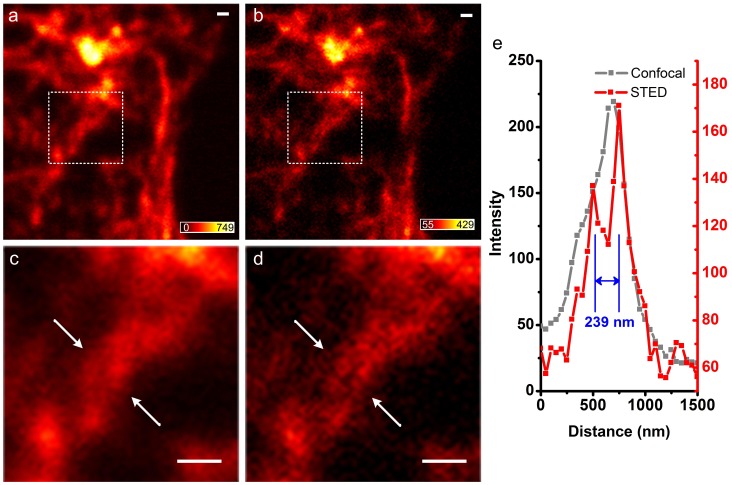
Confocal and corresponding CW-STED images of actin filaments in a HeLa cell. (a) Confocal; (b) CW-STED. The boxed regions in (a) and (b) are magnified in (c) and (d), respectively. The intensities at the cross-section marked in (c) and (d) are plotted in (e). The wavelength and power density for the STED beam is 763 nm and ∼127 MW/cm^2^. Scale bar: 500 nm.

Furthermore, as reported by Oliva et al., insufficient resolution may lead to aliasing of image [32]. In our actin filament imaging of HeLa cell (Figure 6), we observed what appeared to be a single twisted actin filament from the confocal microscopy; this artifact was due to resolution larger than the size (∼8 nm) of filamentous actin and the lateral separation (∼240 nm) between filaments. However, with the aid of CW STED, we can clearly visualize the parallel structure of the actin filaments (Figure 6e).

**Figure 7:**
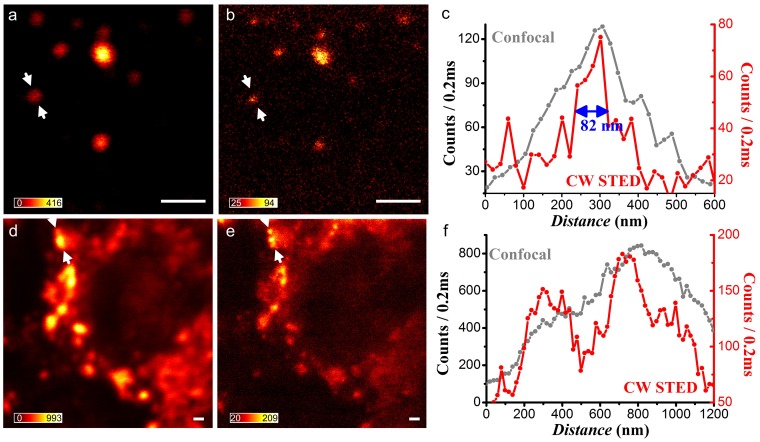
Confocal and corresponding CW-STED images of hRSV genomic RNA granules in a HEp2 cell. Confocal (a) and corresponding CW-STED (b) results of isolated individual RNA, as well as RNA granules images by confocal (c) and CW-STED (d) are shown. The intensity at the cross-section marked in (d) and (e) are plotted in (c, f). The wavelength and power intensity for STED beam is 783 nm and ∼162 MW/cm^2^. Scale bar: 1 µm.

RNA plays a critical role in mediating and regulating gene expression. Understanding RNA localization and dynamics will not only aid in our understanding of native cellular gene expression, but will also provide information on the life cycle of RNA viruses. Given that the size of most RNA molecules is often smaller than the diffraction limit, examining RNA localization is a model problem for the application of CW STED [33]. By utilizing exogenous multiply-labeled fluorescent probes described by Santangelo et al. [34], the genomic RNA of the human respiratory syncytial virus (hRSV strain A2) during cellular infection was specifically labeled with DyLight 650-conjugated probes and imaged via CW STED. As shown in Figure 7, a resolution of 82 nm on STED was achieved comparing to 290 nm on confocal. The contrast in performance is clearly obvious in Figure 7d and e where there is a higher concentration of RNA molecules.

## Discussion

STED nanoscopy has been intensively investigated over the last decade, showing great promise for imaging cellular structures. It utilizes a donut depletion beam to de-excite fluorphoes at the edges of the excitation focus, thus breaking the diffraction limit. It can operate in either pulsed mode or CW mode.The pulsed mode can easily meet the depletion intensity requirements due to the high peak pulsed power, but the precise synchronization between the pulsed excitation laser and pulsed STED laser is both crucial and difficult to achieve and maintain. The successful demonstrations of synchronization-free CW STED, however, employed high power laser or a combination of multiple lasers [21], [22], which are not commonplace, thus limiting STED deployment. We report here a CW STED system with a Ti:Sapphire laser tuned to CW mode, and we achieved a super-resolution of 71 nm laterally, which is only one-tenth the emission wavelength. With super-resolution, the imaging of 200 nm nanospheres demonstrated that beads with distances beyond conventional resolution can be clearly discerned.

Two factors determine the resolution of CW STED nanoscopy: the saturation intensity of the fluorescence dye, and the depletion power applied. Here, for the first time, we have used Dylight 650 for STED nanoscopy and found that its saturation intensity is much lower than that of ATTO 647N, a commonly used STED fluorophore. This might lead to the exploration of other dyes that are more suitable for STED nanoscopy. The depletion power applied relates to two disadvantages associated with CW STED: (1) photobleaching is more pronounced due to constant excitation of the fluorophore and (2) photo-thermal damage to the specimen is possible due to the greater excitation energy applied over time. Therefore, the application of an anti-fade reagent, as well as mounting medium containing an oxygen scavenger, can improve the performance of CW STED imaging. Recent publications have reported that with the addition of Mowiol [21], [35], an oxygen scavenger system [35], [36], and reducing and oxidizing system [35], [37], the photostability of dye molecules can change dramatically. Therefore, it will be important in future studies to further characterize and optimize sample preparation protocols for each dye used in super-resolution nanoscopy..

Here, we have applied CW STED nanoscopy to all three cytoskeleton filaments (microtubule, intermediate filaments, actin filaments), whose sizes are smaller than the resolution of a diffraction-limit microscope, to demonstrate the performance of our STED system, and succeeded in discriminating parallel actin filaments, which appeared as a single structure by confocal imaging. We further demonstrated, for the first time, that STED nanoscopy can discern the Dylight 650-MTRIP-labeled viral RNAs in HEp-2 cells, with a resolution of 82 nm. The imaging capability of CW STED demonstrated in this report should help researchers interested in setting up their own STED systems with access to Ti:Sapphire oscillators, and to further apply STED nanoscopy to a broader, more diverse set of biological problems.

## Materials and Methods

### STED Instrumentation

The STED system was built based on a home-made confocal imaging system with 635 nm excitation. A Ti:Sapphire laser (Griffin-F, KMLabs, USA) pumped by a 6-Watt DPSS 532 nm laser (Finesse-6W, Laser Quantum, UK) was employed as the CW STED source. The output from the laser source is ∼800 mW. To generate the doughnut PSF for effective depletion, a vortex 0–2π phase plate (VPP-A, RPC Photonics) was used. We focused the beams into samples using an oil-immersion objective of NA = 1.4 (100x, PlanAPO, Zeiss). To obtain a 2-D image, a piezo scanning stage (Nanomax, Thorlabs) was employed to move the specimen. A photo-counting avalanche photodiode (SPCM-AQRH-13-FC, Perkin Elmer) was used to collect the fluorescence signal. The scanning and data collection were performed with a DAQ board (USB-6259, National Instruments), and image collection was accomplished with Imspector (Max-Plank Innovation).

### Cells and Viruses

Vero cells (ATCC CCL-81) were maintained in High Glucose DMEM (Lonza) with 10% FBS (Hyclone), 100 U ml-1 penicillin, and 100 µg ml^−1^ streptomycin (Invitrogen). hRSV strain A2 (ATCC VR-1544) was propagated in HEp-2 cells (ATCC CCL-23) at a titer of 1×10^6^ pfu/mL. Cells were plated the day before infection at 25% confluency. Cells were infected by removing the media, washing with PBS (without Ca^2+^ and Mg^2+^), adding virus at a multiplicity of infection (MOI) of 1, and incubating the cells for 1 h at 37°C. After adsorption, fresh medium was added to the inoculum.

### Microtubule Immunofluorescence

Vero’s were plated the day before fixation at 25% confluency and were fixed with 100% methanol for 10 min at −20°C and additionally permeabilized with 100% acetone for 2 min at −20°C. Nonspecific antibody binding was blocked with 5% bovine serum albumin (EMD) in PBS for 30 min at 37°C. Veros were then incubated with a primary antibody against alpha tubulin (rabbit polyclonal IgG, Abcam ab18251) for 30 min at 37°C, washed twice in PBS, and incubated with a secondary antibody (goat anti-rabbit DyLight 650 IgG, Pierce) for 30 min at 37°C, washed twice in PBS, and mounted in a mixture of Mowiol 4–88 (Sigma) and DABCO (VWR) [38].

### Keratin Immunofluoresnce

The PtK2 cells (a gift of Dr. K. Willig) were fixed with ice-cold methanol and blocked with BSA in PBS. The intermediate filaments were stained using indirect immunofluorescence. The primary antibody was a mouse anti-Keratin IgG (Dianova, DLN-09032), and the secondary antibody was a sheep anti-mouse IgG conjugated with ATTO 647N.

### Actin Staining

HeLa cells were maintained in DMEM (Gibco) with 10% FBS (Hyclone). Cells were fixed with 100% methanol for 10 min at −20°C, washed twice in PBS. Cells were then incubated with Atto 647N-Phalloidin (ATTO-TEC) for 1h at room temperature, washed five times in PBS, and mounted in mixture of Mowiol 4–88 (Sigma) and DABCO (VWR).

### hRSV Probe Delivery

To effectively image the genomic RNA of hRSV in HEp-2 cells, MTRIP probes labeled with DyLight 650 were made in a manner similar to [34]. Briefly, 2′-O-methyl RNA/DNA chimeric oligonucleotides, complimentary to the intergenic sites of the hRSV genomic RNA, with a 5′ biotin modification and 5 primary amine-C_6_ deoxy-thymines within the sequence, were labeled with DyLight 650 NHS Ester (Pierce), and filtered using a Zeba spin column (Pierce) to remove free dye. The labeled oligonucleotides were assembled into MTRIP probes and delivered into live cells using streptolysin O (SLO) at 48 h post infection, where they hybridized to multiple sites of the genomic RNA. Pores formed by SLO were closed via recovery in complete growth media for 30 minutes. This was followed by fixation in paraformaldehyde. Cells were mounted in a medium containing 80% glycerol, glucose, glucose oxidase, catalase, and β-mercaptoethanol [35].
